# Are patients willing to help reduce contrast material in the environment? The outpatient use of urine bags after contrast-enhanced computed tomography

**DOI:** 10.1007/s00330-025-11565-6

**Published:** 2025-04-11

**Authors:** Helena M. Dekker, Hendrik Bram Beltman, Mathias Prokop

**Affiliations:** Department of Medical Imaging, Radboud University Medical Center, Nijmegen, The Netherlands

**Keywords:** Contrast media, Urine, Sustainable development, Environmental exposure, Computed tomography

## Abstract

**Objectives:**

We studied the willingness of outpatients to reduce the environmental impact of contrast media by using urine bags to collect excreted contrast material after contrast-enhanced computed tomography (ceCT).

**Materials and methods:**

In this prospective single-center cohort study, we provided consecutive outpatients undergoing ceCT with information about contrast material excretion. We then offered urine bags to collect their urine for the first four consecutive urination sessions after the ceCT examination. An absorbent pad within these bags transforms the urine from liquid to solid so that the bags can be disposed of via the household waste system to avoid water contamination. Participants were asked to complete a questionnaire-based telephone interview after ceCT.

**Results:**

Of the 671 consecutive outpatients undergoing ceCT, 503 patients (75%) participated in the study, of whom 476 participants (mean age, 63 years; range, 20–88 years) successfully underwent a telephone interview. 455 of 476 patients (96%) had used at least one urine bag; 84% had used 3 or 4 urine bags. Time between ceCT and use of the final urine bag averaged 9.46 h (range, 0.1–28.5 h). 434 patients (91%) were “willing to collaborate on solutions to reduce contrast agent residues in water.” 380 patients (80% of participants) stated to “definitely use the urine bag again after my next ceCT scan.”

**Conclusion:**

Most outpatients were willing to use urine bags after ceCT. Urine bags were used for an average of 9 h after ceCT, ensuring that a substantial amount of the administered contrast medium does not enter the sewage system.

**Key Points:**

***Question*** *It is not known whether there is enough willingness among patients to use urine bags after contrast-enhanced CT (ceCT) to reduce contrast material in the water supply*.

***Findings*** *Urine bags offer a viable option to reduce the environmental impact of contrast agents and are acceptable to most outpatients*.

***Clinical relevance*** *The majority of outpatients were willing to help reduce the environmental impact of contrast material by using urine bags after ceCT so that excreted contrast material does not enter the water supply*.

**Graphical Abstract:**

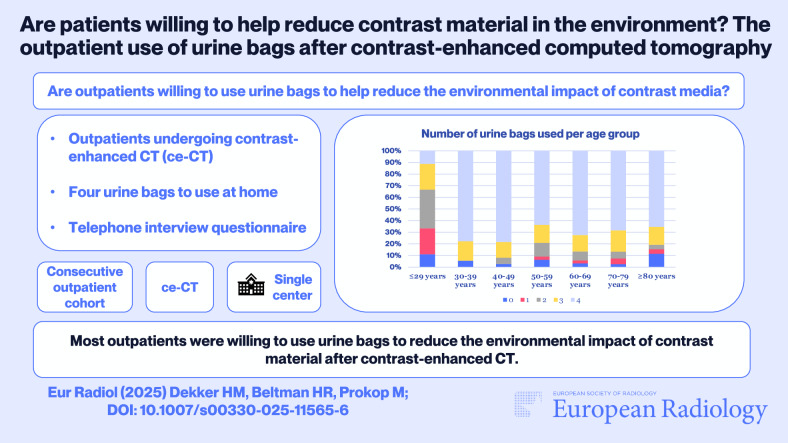

## Introduction

Iodinated contrast media (ICM) are necessary for diagnostic imaging in contrast-enhanced CT scans. The use of ICM is steadily increasing due to the rise in CT scanner availability and the need for more medical treatment options that require a CT scan. More than 10 million liters of ICM are used per year worldwide [1].

However, ICMs are increasingly present in surface water and sources of drinking water, and there is emerging evidence that ICM breakdown products are toxic [1]. Commonly used drinking water purification techniques are often ineffective in removing ICM, which can lead to the formation of toxic iodinated disinfection byproducts [2]. Some ICM are linked to biotransformation under aerobic and anaerobic conditions, which occur in municipal wastewater treatment plants (an/aerobic) and activated sewage sludge (anaerobic) [3, 4]. Advanced oxidation techniques with chlorine, ozone, chlorine dioxide, or chloramines are used during drinking water production for the removal of persistent contaminants and for disinfection. Such oxidation techniques cause the formation of iodinated byproducts that tend to be more (geno)toxic than non-iodinated byproducts [5, 6]. The problem of ICM in drinking water preparation has so far not yet been solved. To address this issue, all parties involved need to cooperate, from the producer of contrast medium to the patients, the ultimate consumers of drinking water.

**Fig. 1:**
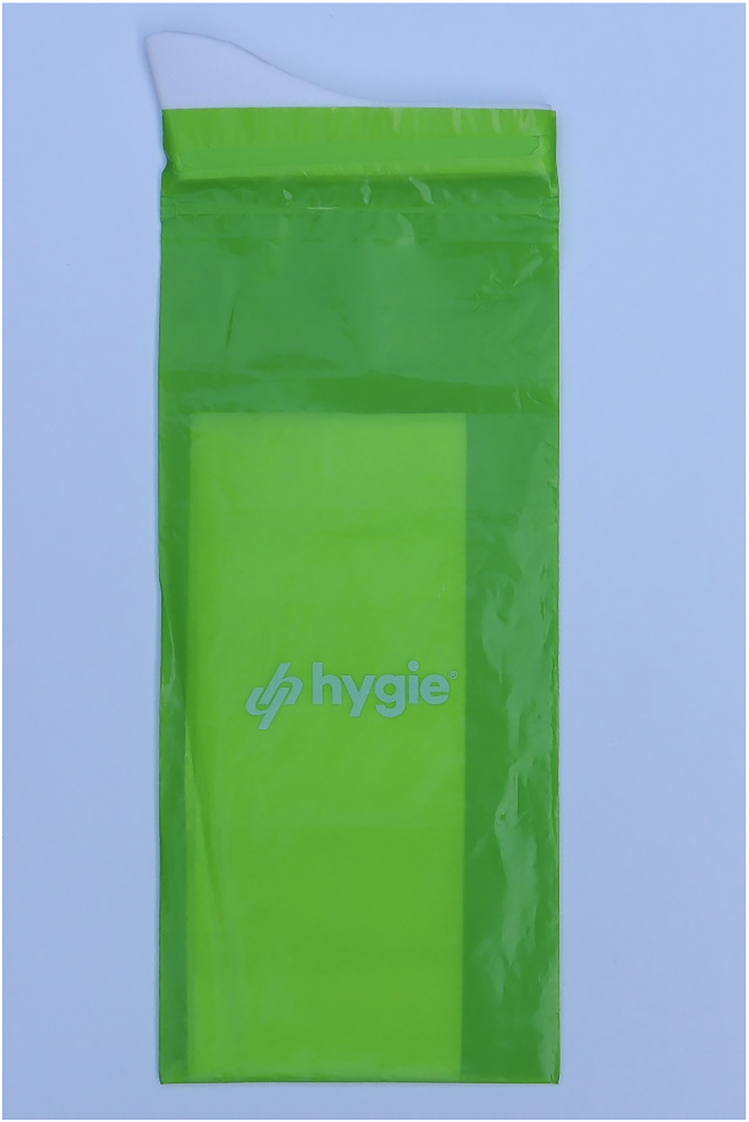
Urine bag

Radiologists can implement measures to optimize the use of contrast media, reduce contrast waste and collect unused contrast material at the point of application [1]. Contrast media waste management can be further optimized by collecting contrast agent residues separately. They then can be disposed of via hospital channels, or the uncontaminated leftovers can be returned through collection services offered by producers [7–9]. These measures make sure that only the minimally necessary amount of contrast material is used. However, these measures must be amended by methods to collect and remove contrast-containing urine before it enters the water supply. One potential method is the use of urine bags to collect urine after contrast-enhanced imaging procedures (Fig. 1). These urine bags contain an absorbent pad that transforms liquid urine into a solid substance that can be disposed of via solid household waste. Burning this waste will return the iodine bound in the contrast material into naturally occurring iodine and iodine salts, which are normally present in nature as well [10].

First results of this technique have been reported in the *Merkmal* study [11] and the *MIndER* study [12] in Germany and the *Brede Plaszakkenproef* in the Netherlands [13]. However, it is unknown how many patients would be willing to use such a urine bag in an outpatient setting, how many of these bags would be needed per patient and which time interval after the CT scan would on average be covered to collect excreted contrast.

This paper reports the results of a survey on the attitudes of a cohort of outpatients toward collaborating on solutions to reduce contrast agent residues in water. It further studies crucial information needed for the practical implementation of such a method.

## Materials and methods

The local review board of the Radboud University Medical Centre (Radboudumc) approved this prospective study (IRB number: 2022-15777). All patients provided written informed consent.

### Participants

Patients scheduled for contrast-enhanced CT at the Department of Medical Imaging at Radboudumc were selected from the working list of one of the CT scanners used for outpatient services. A separate waiting room is available at this CT scanner. The inclusion criteria were: adult patients (≥ 18 years); outpatients; no examinations requiring radioactive material on the same day; electively scheduled for a contrast-enhanced CT scan; good enough comprehension of the Dutch language to be able to understand the informed consent form and the questionnaire. Participation was on a once-only basis.

### Questionnaire

The data were collected by means of telephone questionnaires. These contained seven questions concerning the use of urine bags, time of application of the final urine bag, discarding the urine bags, education level, and attitude about collaboration on solutions to reduce contrast agent residues in water, and the use of the urine bag after their subsequent ceCT scan. Most questions were adapted from a questionnaire, developed by a working group of the Dutch Brede Plaszakkenproef [13].

### Procedure for data collection

Consecutive patients meeting the selection criteria were asked to participate in the cohort study. The patients were recruited over an 8-week period in 2023. This study was conducted by a small group of students who informed patients, obtained informed consent, and collected all data.

The patients were informed about the study, both verbally and in writing (information folder). We provided information about contrast material excretion and environmental impact. The process of informing the patient about the use of urine bags was supported by a video. This video was shown on a large screen in the waiting room near the CT scanner [14]. Participants signed an informed consent form.

We offered patients four urine bags to collect their urine for the first four consecutive urination sessions after the ceCT examination. A leaflet with information on how to use the urine bags was included.

Patients were requested to complete a telephone questionnaire following the use of urine bags. They were also asked to provide a telephone number where they could be contacted. Additionally, a hard copy questionnaire was distributed to all selected patients. The day after the ceCT examination, a student contacted patients to review the questionnaire. If patients were not reachable initially, they were contacted again later.

In addition to the telephone questionnaire data, the following data were also collected: patient’s date of birth, gender, date and time of contrast agent administration for the ceCT. All data was processed anonymously.

### Urine bags

The urine bags (SA-HYGI URIO-000) used in this study were produced by Hygie Canada Inc. and distributed by Dispocare. The urine bags consist of a significant proportion (74%) of recycled materials [15].

The bags contain an absorbent pad that fixes the urine. They are sealable (Fig. 1) and are to be discarded via the general household waste disposal system. The urine bags were acquired using a grant provided jointly by the Sustainability Board of Radboudumc and the Dutch Healthcare Insurance Company VGZ.

### Data analysis

Data was analyzed using IBM SPSS Statistics (Version 29). Analysis of the data was undertaken using descriptive statistics, and the results were reported as frequencies and percentages. A Chi-square test was used to evaluate the differences between the sexes for the use of urine bags.

### Response

Of 671 consecutive outpatients undergoing ceCT, 503 patients (75%) were willing to participate in the study and use urine bags. The telephone questionnaire was successfully obtained from 476 participants (95%).

## Results

A total of 476 participants (mean age, 63 years; standard deviation 12.6, range, 20–88 years; 265 men) were enrolled in the study. The demographic characteristics of these 476 participants are presented in Table 1.

**Table 1:** Demographic data of the survey participants

Demographic characteristic	*n*	%
Sex		
Female	211	44.3
Male	265	55.7
Age		
< 30	9	1.9
30–39	18	3.8
40–49	37	7.8
50–59	110	23.1
60–69	156	32.7
70–79	120	25.2
≥ 80	26	5.5
Urine bag use		
4	326	68.5
3	75	15.7
2	38	8.0
1	16	3.4
0	21	4.4

The vast majority, 455 of 476 patients (96%), had used at least one urine bag; 69% had used 4 urine bags, 16% had used 3 urine bags, 8% had used 2 urine bags, and 3% had used 1 urine bag. Twenty-one patients (4%) did not use the urine bags. Reasons for not using urine bags were that patients had an excessive need to urinate, forgot or found it too much of a hassle.

In this study, 1904 urine bags were distributed to the 476 patients participating in the telephone interview. Of these bags, 283 (15%) remained unused.

**Fig. 2:**
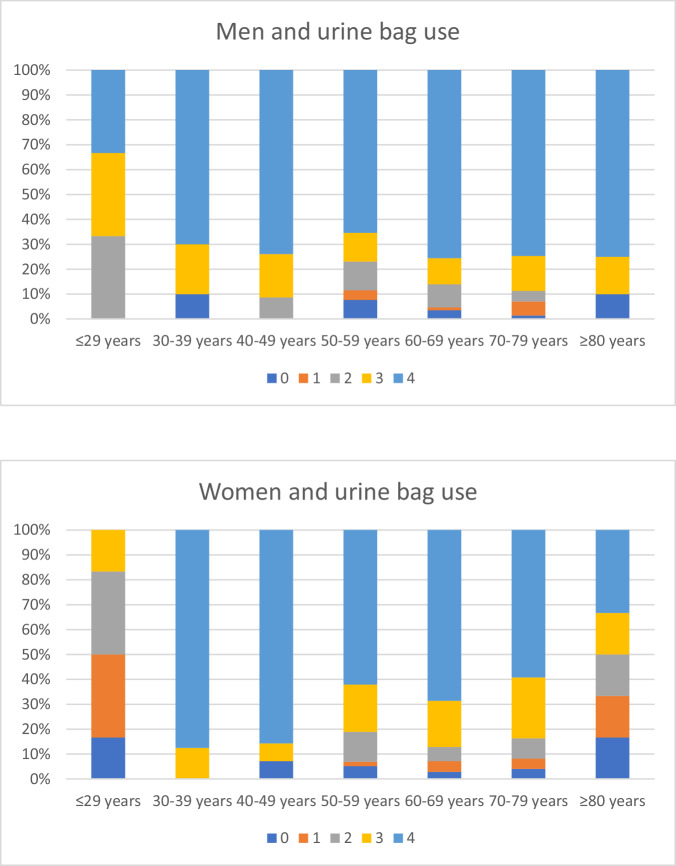
The use of urine bags by age group and sex

There was no difference in the overall use of urine bags between male and female participants: 96% of the male participants and 95% of the female participants used 1–4 urine bags. However, a significantly larger proportion of men (72%) used 4 urine bags than women (64%; Chi-square test, *p* < 0.05). Figure 2 shows the use of urine bags by age group and sex.

The highest user rate of 3–4 urine bags among men was found in the 40–49 year age group. In women, this was found in the 30–39 year age group. The lowest user rates of 3–4 urine bags were in the ≤ 29 age group for both sexes, among men in the 50–59 age group and for women in the 80+ age group.

The distribution of education level is presented in Table 2. The highest use of 1–4 urine bags was found in the secondary education group with an average age of 65.6 years. The lowest rate was seen in the primary or non-education group. However, these differences were not statistically significant.

**Table 2:** Education level of the survey participants

Education level	*n*	% 1–4 urine bags used	Average age (range)	Sex (*n* = male)
Primary education/non	20	90%	64.1 (35.9–84.8)	11
Secondary education	105	98%	65.6 (30.6–87.7)	55
Secondary vocational education	164	96%	61.2 (19.6–84.8)	93
Higher vocational education	118	95%	62.8 (25.5–87.4)	64
Academic education	59	97%	61.3 (31.2–83.8)	40
Not given	10	70%	65.4 (51.9–80.1)	2

The time between the administration of the contrast agent for the ceCT and use of the final urine bag was on average 9.46 h (range, 0.10–28.53 h), for the group of participants. Table 3 shows the time between contrast agent administration and use of the final urine bag, with distinction per group by total number of urine bags used.

**Table 3:** Time between contrast agent administration and use of the final urine bag, with distinction per group by total number of urine bags used

Urine bags used	Average hours	Minimum	Maximum
1	02:47	00:10	07:32
2	07:46	00:41	20:00
3	10:51	01:31	28:53
4	10:06	01:42	28:40
1–4	09:46	00:10	28:53

Unaided use of urine bags was possible by 445 patients, accounting for 98% of the patients who used the urine bags. There was no difference in the overall use of urine bags between patients who required assistance and those who did not.

Most participants discarded the urine bags via general household waste disposal at home (66%) or at other locations (32%). A few patients used the first urine bag at the hospital and disposed of it in a residual waste container in the hospital.

The participants were asked whether they agreed with two statements about their attitude to collaborate on solutions to reduce the environmental impact of contrast agents. The vast majority of those patients who responded to the telephone questionnaire (434/476 = 91%) were “willing to collaborate on solutions to reduce contrast agent residues in water.” 380 patients stated to “definitely use the urine bag after my subsequent ceCT scan.” This represents 80% of the study participants and 57% of all outpatients (*n* = 671) invited to participate in the study. A summary of the results is presented in Table 4.

**Table 4:** Participants indicated their agreement with two statements, with distinction to number and percentage of participants

Statement	Agree	Somewhat agree	Somewhat disagree	Disagree	No opinion
As a patient, I am willing to collaborate on solutions to reduce contrast agent residues in our water	434 (91%)	26 (5%)	1 (< 1%)	5 (1%)	7 (2%)
I will definitely use the urine bag after my subsequent ceCT scan	380 (80%)	42 (9%)	10 (2%)	29 (6%)	13 (3%)

## Discussion

In this prospective cohort study, most outpatients (75%) were willing to use urine bags after ceCT. Urine bags were used for an average of 9 h after ceCT ensuring that a substantial amount of the administered contrast medium is excreted. The use of urine bags ensures that the excreted contrast material will not enter the sewage system but is disposed of with the general household waste. The overall use of urine bags was highest among patients aged 30–80 years. Men slightly but significantly more often use 3–4 urine bags instead of 1–2 bags. No significant differences were observed based on education level.

Three previous projects with urine bags have been described in the literature. In Germany, a pilot project with urine bags was performed in 2200 patients [11]. The patients received a package with four urine bags and instructions for use. The vast majority of patients (87%) indicated that they had used the urine bags. In the MindER pilot project, patients were provided with six urine bags for home use [12], of which 20–25% of the pilot participants completed a questionnaire. On average, participants used 5.4 urine bags each. More than half of the patients used the urine bags for half a day or longer, and over 75% of those who completed the questionnaire expressed their willingness to use the urine bags in the future.

In the Netherlands, a pilot study was carried out in six hospitals, during the period from November 2020 to March 2021. A total of 9394 outpatients received three or four urine bags after a contrast-enhanced CT. Patients had to log into a website themselves to complete the self-administered questionnaire. Only 16% of the participants completed the online questionnaire [13], making it impossible to judge how often the urine bags were actually used in the total cohort. Most responding patients (60%) indicated that they would like to contribute to a better environment. Among those who filled in the questionnaire, 1471 patients (99%) had used at least one urine bag; 85% had used 3 or 4 urine bags. These results are broadly consistent with our cohort study. However, there is no information available regarding the duration of use for urine bags or about the education levels of patients.

In patients with good renal function, the contrast medium is excreted over 24 h [16]. The elimination half-life increases progressively with increasing renal impairment [16]. In patients with mild and moderate renal impairment, approximately 50% of the injected contrast medium was recovered in urine between 4 and 8 h [16]. Our study population includes many elderly patients with a variety of conditions, including many oncological conditions, so we can expect patients’ renal function to be variable. This suggests that the average of 9 h which urine was collected in our study should remove even more than 50% of the injected contrast material, thus making sure that it will not enter the sewage water system.

The effect of using urine bags on the concentration of ICM in the River Ruhr in Germany was studied in the MERK’MAL project. The results from this pilot study showed a reduction in ICM concentration compared to the baseline concentration measured at the same sampling point in the effluent of the corresponding wastewater treatment plant. The mean ICM reduction ranged between 20 and 34%; median reduction of ICM concentrations ranged between 7 and 33% [17].

The urine bags are disposed of via the domestic waste system. If this domestic waste is further processed in an incinerator, the contrast agent is reduced to naturally occurring iodine and iodine salts [10], which are not known to have a negative environmental impact. Urine bags and ICMs disposed of in landfills should also have no negative environmental impact, provided the landfill is properly managed. Physical barriers such as clay or synthetic liners and/or collection and treatment of landfill leachate will prevent contamination of the surrounding groundwater. In the absence of such measures, other landfill-related contaminants will pose a much greater threat to the environment than ICMs.

Incineration of ICM can give rise to hazardous “purple smoke” (high concentration of I_2_ molecules in the air), but only if high quantities of ICM waste are incinerated. Urine bags in household waste represent a minuscule portion of the whole waste volume and therefore should not induce this phenomenon. This was confirmed by an environmental impact study commissioned by the Dutch Ministry of Infrastructure and Environment in 2016, which estimated that the impact from household waste incineration would be negligible [10]. However, if iodinated waste is declared as hazardous waste, like in Switzerland, and is collected separately, those larger iodine quantities will need incineration with special requirements. The advantage of collecting iodinated waste, however, is the option for recycling the collected iodine. Finally, any I_2_ molecules released into the air, into fly ash or into wastewater will ultimately be converted into innocuous iodine ions upon contact with water.

The urine bags used in this study contain a significant proportion of non-toxic recycled materials to minimize their environmental impact. However, the production, transportation and incineration of these bags contribute to their overall environmental footprint. While this has not been studied in detail, the environmental impact can be assumed small relative to the toxic byproducts of contrast material in our water supplies. Costs for urine bags are low but become large if multiplied by the total number of eligible procedures. The decision of who will pay will be a political one unless environmentally conscious patients choose to pay for these measures themselves.

Beyond urine bags, there are other methods for separately collecting contrast agents excreted in urine. In the Greenwater Project [18], patients were asked to extend their hospital stay by up to 60 min to collect their initial urine in dedicated canisters. This approach achieved a recovery rate of 51.2% for iodinated contrast media (ICM) and 12.9% for gadolinium-based contrast agents (GBCA).

An alternative is the use of separation toilets. In Germany, a pilot study with a separation toilet and a feedback system showed 16% patient use without encouragement [19]. In the Netherlands, a cardiology department pilot replaced a standard toilet with a separation toilet using absorbent cartridges that bind ICM. With its 100% removal efficiency of contrast agents excreted during post-procedure stays, this system opens the potential for iodine recycling [20].

While raw materials for contrast material production become increasingly scarce globally, initiatives to recycle iodine [7–9, 21] or gadolinium [22] from collected CMs are still in their infancy. The use of urine bags offers the advantage of allowing patients to continue collecting contrast agents at home and currently remains the only viable method outside of the hospital setting for preventing contrast agents from entering wastewater systems.

The major strength of our prospective cohort study lies in our ability to achieve a response rate of 95%. This high response rate was possible because patients were contacted by telephone, and our student interviewers went through the questionnaire with them, making sure they understood the questions and provided adequate responses.

We provided a video in the waiting room and an information folder to raise awareness about contrast material in our water supplies and to optimize participation in this study. There was also a student present in the waiting room, who could provide active verbal explanation and give patients the opportunity to ask questions. With this approach, we achieved a participation rate of 75% among those patients scheduled for an outpatient ceCT exam at our university department.

During the *Brede Plaszakkenproef* [13], urine bags were distributed to patients by radiographers. After the trial, the radiographers were asked how much additional time it took to distribute the urine bags. Of those surveyed, 70% indicated that it required little to no extra time. However, it is essential to ensure proper information is provided to patients through informational brochures and a short instructional video to streamline the process.

Our study has limitations. Specifically, 5% of the participants could not be reached to complete the questionnaire. Additionally, the study only included outpatients at one specific CT scanner dedicated to outpatient services, and not all outpatients scanned also at other scanners during the study period. The fact that we had a dedicated person who could provide information and could be approached for questions may have also increased the participation rate relative to a setting where this is not possible or would have to be done by radiographers.

With the growing emphasis on more environmentally friendly medicine, our profession needs to actively work on reducing its environmental impact. To tackle the impact of contrast media, not only radiologists but also patients can contribute. The use of urine bags is one of those measures: it has the potential to significantly reduce the amount of contrast media in wastewater, and the vast majority of patients are willing to play their part in it.

## Abbreviations

ceCT: Contrast-enhanced computed tomography
ICM: Iodinated contrast media
Radboudumc: Radboud University Medical Centre
